# APSified Peripapillary Vessel Density in Glaucoma Suspects and Open-Angle Glaucoma

**DOI:** 10.3390/diagnostics16060932

**Published:** 2026-03-21

**Authors:** Michael Moritz, Julia Schottenhamml, Marius Muenk, Meike Müller, Christian Mardin, Bettina Hohberger

**Affiliations:** Department of Ophthalmology, Uniklinikum Erlangen, Friedrich-Alexander-Universität Erlangen-Nürnberg, 91054 Erlangen, Germany; julia.schottenhamml@uk-erlangen.de (J.S.); marius.muenk@gmx.de (M.M.); mk.mueller@web.de (M.M.); christian.mardin@uk-erlangen.de (C.M.); bettina.hohberger@uk-erlangen.de (B.H.)

**Keywords:** glaucoma, OHT, glaucoma suspects, OCT-A, vessel density

## Abstract

**Background/Objectives**: Optical coherence tomography-angiography (OCT-A) is a non-invasive method of visualizing the capillary system. As vascular dysregulation impacts glaucoma pathogenesis, the aim of this study was to evaluate APSified-BMO-based-peripapillary vessel density (VD) in patients with ocular hypertension (OHT), pre-perimetric-open-angle glaucoma, as well as primary (POAG) and secondary (SOAG) open-angle glaucoma in comparison to healthy controls using OCT-A. **Methods**: The present study included 180 eyes from 115 patients of the Erlangen Glaucoma Registry, divided into 35 eyes with OHT, 16 pre-perimetric-OAG eyes, 64 OAG eyes—which were subdivided into 37 POAG and 27 SOAG eyes—and 65 healthy controls. All subjects underwent measurements of the retinal nerve fiber layer (RNFL), inner nuclear layer (INL), retinal ganglion cell (RGC) layer, and Bruch membrane opening–minimum rim width (BMO-MRW). APSified-BMO-based-peripapillary vessel density (VD) was visualized by using OCT-A and quantified using the Erlangen Angio Tool. **Results**: Mean APSified-BMO-based peripapillary VD showed a significant correlation with age (*p* < 0.0001). Considering the age effect, mean APSified-BMO-based peripapillary VD of OAG was significantly lower compared to healthy eyes (*p* < 0.0001) and OHT (*p* = 0.016). Subgroup analysis yielded a significant difference in mean APSified-BMO-based peripapillary VD between controls and POAG (*p* = 0.001) and SOAG (*p* = 0.018), respectively. In addition, a significant difference was observed between OHT and POAG patients (*p* = 0.036). No significant differences were observed between the OHT, pre-perimetric-OAG, and healthy eyes, respectively. **Conclusions**: As peripapillary VD was significantly decreased in glaucoma patients compared to controls, the data might suggest that peripapillary VD might be useful for monitoring glaucoma progress.

## 1. Introduction

Glaucoma is one of the leading causes of blindness in the developing countries [1]. This neurodegenerative disease, defined by a typical pathological change in the optic nerve head with corresponding perimetric field loss, is estimated to affect more than 79 million people worldwide [2]. If about 40% of retinal ganglion cells are lost, clinical symptoms will be recognized by the patients themselves: the visual field will become restricted and may even result in complete blindness [3,4,5]. Thus, an early diagnosis is recommended in order to win sighted lifetime. In addition to its main risk factor, the pathological increased intraocular pressure (IOP), various factors such as oxidative stress, autoimmune phenomena, and genetic susceptibility can contribute to this complex interplay, though they have yet to be fully elucidated up to now [6,7,8,9,10,11]. In addition, vascular dysfunction contributes to glaucoma pathogenesis [12,13,14,15]. The ocular blood flow (OBF) is a complex system regulated by several factors (e.g., autonomic nervous system) in order to support the demand for oxygen and nutrition in each ocular structure [16]. Thus, vascular dysfunctions denominate the discrepancy of local demand and supply, being a sign of ‘dysregulated regulation’ of veins, arteries, and capillaries (as defined by over- and underperfusion) [17]. Several systemic factors (e.g., inflammatory disorders (such as systemic lupus erythematosus), endothelium-derived vasoactive factors) interact within the pathogenetic dysregulated OBF, affecting a wide range of vessels (e.g., the choroid, retrobulbar vessels, retinal capillaries) [18,19,20,21].

**Figure 1:**
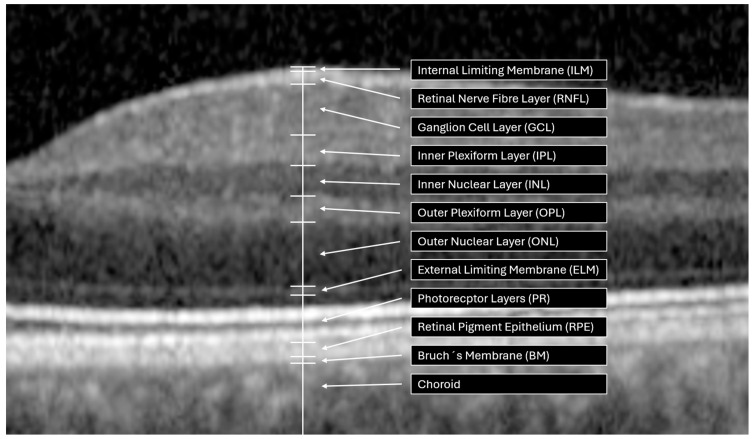
Optical coherence tomography (OCT) retinal layer annotation from a high-resolution OCT image of a control patient.

**Figure 2:**
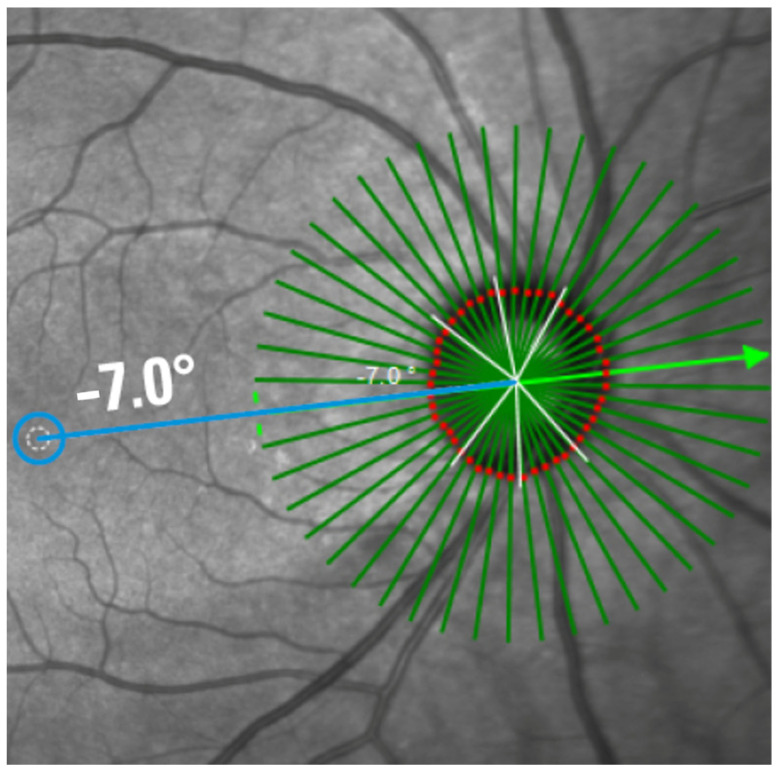
Alignment of the Bruch’s membrane opening base scan to the Fovea–Bruch’s membrane opening (BMO) centerline using the anatomical positioning system (APS). The 24 green scan lines are automatically generated by the Glaucoma Module Premium Edition (GMPE, Heidelberg Engineering GmbH, Heidelberg, Germany) and detect 48 Bruch membrane opening (BMO) positions along the optic nerve head, determining the BMO-based disc margins (red dots). The blue line shows the axis between the fovea and the centre of the BMO. The green arrow shows which scan line is currently selected (courtesy of Heidelberg Engineering GmbH, Heidelberg, Germany).

Beyond conventional assessments (e.g., IOP measurement, fundoscopy, and perimetry) optical coherence tomography (OCT) serves as a non-invasive and highly precise imaging technique for diagnosis and longitudinal monitoring of glaucoma [22]. This technique enables measurements of the thickness of distinct retinal layers, including the retinal nerve fiber layer (RNFL), the inner nuclear layer (INL), the retinal ganglion cell layer (RGCL), and the minimum rim width of the Bruch’s membrane opening (BMO-MRW, Figure 1). Based on OCT technology, OCT-A has been refined for use in visualization of the vascular network in the macular and peripapillary regions [23,24]. Using OCT-A, fast and high resolution of 3-dimensional images of retinochoroidal microcirculation can be obtained without the injection of dye, as with fluorescence angiography. For this purpose, OCT-A uses a motion contrast algorithm to detect moving blood cells, resulting in a temporal difference reflection due to the movement of blood cells in the same region. One vascular biomarker, based on OCT-A scans, is the vessel density (VD), quantified as percentage of white (i.e., movement) to black (i.e., no movement) pixels in the scanned area. Previous studies have already shown that the peripapillary VD in glaucoma eyes is significantly lower compared to healthy eyes, correlating with severity of glaucoma [25,26,27,28,29]. The diagnostic ability of VD increases with increasing disease progression [30]. Recent studies suggest that the dysregulated retinal capillary system (quantified by OCT-angiography, OCT-A) might even show glaucoma progression earlier compared to neuronal changes, quantified by OCT [31]. To the best of our knowledge, all recent studies on peripapillary VD were performed by using a ring-shaped or elliptical-shaped region of interest (ROI). This ring-shaped ROI extends from the edges of the optic nerve head (ONH) and has varying diameters [32,33,34]. Accordingly, the peripapillary VD also varies depending on the ROI. As the elliptical rings are only an approximation, and the margin of the ONH varies from individuals, each analysis of the peripapillary VD must be adapted to the shape and size of the ONH in order to quantify the real microvascular bed, adjacent to the ONH. An anatomical ocular landmark represents the BMO, defined as the peripapillary end of the retinal pigment epithelial (RPE) layer. Representing the actual position of the optic disk as its best [35], BMO-based analyses measured using spectral domain OCT (SD-OCT) thus outperform rim-based analyses visible using fundoscopy [36]. Considering each individual anatomy, the anatomical positioning system (APS, part of the Glaucoma module premium edition (GMPE), Heidelberg Engineering) was introduced: APS adapts each individual retinal characteristic to a baseline reference line. This reference line is the tie line between the center of the BMO and the center of the Fovea (i.e., FoBMOC axis). Implementation of APS enables APSified-OCT-A scans, defined by aligning OCT-A scans to each patient’s individual Fovea-to-Bruch’s membrane center (FoBMOC) axis (Figure 2), which ensures that all scans are aligned according to the anatomical arrangement of the axons in each eye, ensuring reliable point-to-point comparisons for all OCT-A scans, to avoid fluctuations in VD due to location-specific variations. This can improve individual regional VD allocation. Thus, the aim of the present study was to evaluate APSified-BMO-based peripapillary VD in glaucoma suspects (ocular hypertension, OHT; pre-perimetric open-angle glaucoma), as well as patients with primary (POAG) and secondary OAG (SOAG) compared to a control cohort. For this purpose, two models were created. The first model incorporated the control group, glaucoma suspects (OHT, pre-perimetric open-angle glaucoma), and the entire OAG cohort. In the second model, the OAG was further subdivided into the POAG and SOAG. Furthermore, the BMO-MRW and the RNFL thickness of these patient cohorts were analyzed.

## 2. Material and Methods

### 2.1. Patients

A total 180 eyes of 115 probands from the Erlanger Glaucoma Registry (Erlangen Glaucoma Register, ISSN 2191-5008, CS-2011; NCT00494923 [37], Department of Ophthalmology, University of Erlangen) and local residents were recruited, comprising 35 eyes with OHT, 16 eyes with pre-perimetric OAG, 64 eyes with OAG (subdivided into 37 eyes with POAG; 27 eyes with SOAG), and 65 healthy controls. The demographic data are shown in Table 1.

Each of the subjects underwent an ophthalmologic examination including fundoscopy, slit-lamp biomicroscopy, and measurement of the IOP by Goldmann applanation tonometry (Haag-Streit AG, Köniz, Switzerland). Additionally, measurements of the BMO-MRW and RNFL, RGCL, and INL thicknesses were performed (Heidelberg OCT II Spectralis, version 1.9.10.0, Heidelberg Engineering, Heidelberg, Germany). The RNFL thickness was divided into three concentric rings, with a diameter of 3.5 mm for the inner ring, 4.1 mm for the middle ring, and 4.7 mm for the outer ring. The significance of the larger rings becomes apparent when the inner circle cannot be interpreted due to an influencing pathology. Perimetric field loss was measured by white-on-white perimetry (Octopus 500, Interzeag, Schlieren, Switzerland; program G1). This study was approved by the local ethics committee and was performed in accordance with the tenets of the declaration of Helsinki.

The patients of each group were selected based on the following inclusion criteria:

#### 2.1.1. Control Cohort

The healthy subjects showed no ocular disorder or systemic disease with ocular involvement. Exclusion criteria included pregnancy, the lactation period, and mental incapacity to participate in the study.

#### 2.1.2. OHT

The OHT patient had a normal visual field, quantified by standard white-on-white full-field perimetry (Octopus 500, G1 protocol, Interzeag, Schlieren, Switzerland). In accordance with the Jonas classification, the patient did not show any glaucomatous damage to the optic disk [38]. IOP measurements obtained via Goldmann applanation tonometry (repeated twice) exceeded 21 mmHg.

#### 2.1.3. Pre-Perimetric OAG

The pre-perimetric OAG patient had a normal visual field, as quantified by standard white-on-white full-field perimetry (Octopus 500, G1 protocol, Interzeag, Schlieren, Switzerland). In accordance with the Jonas classification, classified according to the pathognomonic decreased neuroretinal rim [38], the patient showed a glaucomatous altered optic disk as well as an open anterior chamber. The following characteristics were identified as clinical signs of glaucomatous optic disk atrophy by Jonas et al., as outlined in the following guidelines: vertical elongation of the optic disk, indentation of the neuroretinal margin and thinning in the lower region, as well as optic disk hemorrhages and focal loss of visibility in the nerve fiber layer [39]. IOP measurements obtained via Goldmann applanation tonometry (repeated twice) exceeded 21 mmHg.

#### 2.1.4. POAG

The POAG patient had visual field defects as shown by standard white-on-white full-field perimetry (Octopus 500, G1 protocol, Interzeag, Schlieren, Switzerland). In accordance with the Jonas classification, the patient showed a glaucomatous altered optic disk and an open anterior chamber [38]. IOP measurements obtained via Goldmann applanation tonometry (repeated twice) exceeded 21 mmHg.

#### 2.1.5. SOAG

The SOAG patient showed visual field defects as measured by standard white-on-white full-field perimetry (Octopus 500, G1 protocol, Interzeag, Schlieren, Switzerland). In accordance with the Jonas classification, the patient had a glaucomatous altered optic disk and an open anterior chamber [38]. Furthermore, SOAG is diagnosed based on the presence of melanin dispersion in the anterior chamber, and/or Krukenberg spindle formation (melanin dispersion glaucoma), as well as the presence of PEX material on the anterior chamber structures (PEX glaucoma) [39]. IOP measurements obtained via Goldmann applanation tonometry (repeated twice) exceeded 21 mmHg.

### 2.2. Morphometric Measurements

Spectral domain optical coherence tomography (Spectralis OCT version 1.9.10.0, Heidelberg Engineering, Heidelberg, Germany) was used to perform morphometric measurements such as thickness of the inner, middle, and outer rings of the RNFL and the BMO-MRW.

#### OCT-A

**Figure 3:**
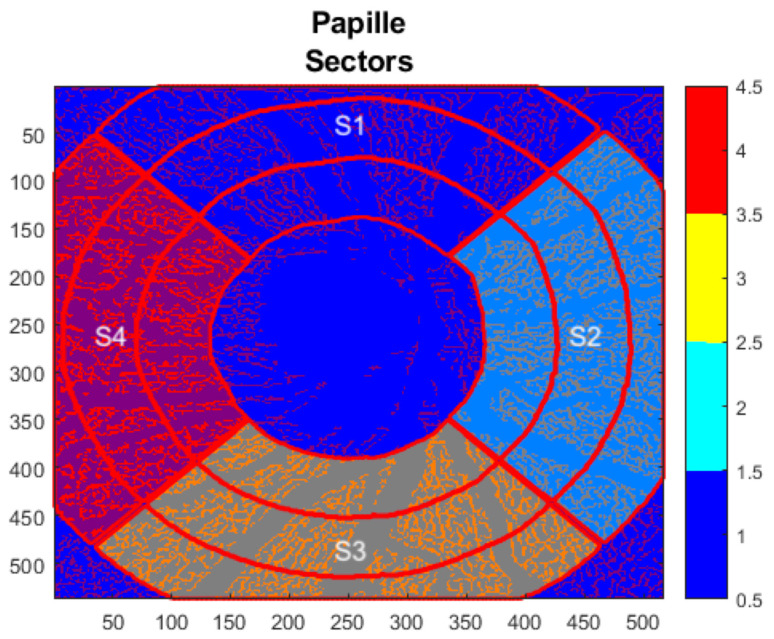
Analysis of peripapillary vessel density with anatomical boundary of Bruch’s membrane opening by the Erlangen Angio Tool (version 2.0 coded in Matlab (The MathWorks, Inc., Natick, MA, USA, R2017b)). S1 represents the superior sector, S2 the nasal sector, S3 the inferior sector, and S4 the temporal sector.

OCT-A images were obtained using Heidelberg OCT II spectral imaging (Heidelberg Engineering, Heidelberg, Germany). The scans have a size of 2.9 mm × 2.9 mm with an angle of 15° × 15° and the highest possible resolution of 5.7 µm/pixel. En face projections starting from the inner limiting membrane (ILM) to 150 µm below were automatically computed by the manufacturer’s software. The data was then exported and analyzed using the EA Tool (version 2.0 coded in Matlab (The MathWorks, Inc., Natick, MA, USA, R2017b), which uses the Frangi vesselness filter (scale range: [1, 10], scale ratio: 2, beta1: 0.5, beta2: 15) and the Otsu thresholding algorithm (threshold automatically determined using Matlab’s built-in function) for generating binarized vessel maps of the en face OCT-A images [40]. The EA tool allows quantification of VD with a high degree of reliability and reproducibility [41]. The analysis of the scans was BMO-based, meaning that instead of using annuli of different sizes, the anatomical boundary of the ONH (the BMO boundary) is used to define the area to quantify. The BMO boundaries were determined using the Glaucoma module premium edition (GMPE) software, which automatically detects 48 BMO positions along the optic nerve head within 24 scan lines to determine the BMO-based papillary margin. Following the acquisition of the scan, the BMO segmentation was examined by navigating through the OCT scans and evaluating the ILM segmentation and the clearly identifiable BMO points. If necessary, adjustments were made to ensure the accuracy of the segmentation. Moreover, the anatomical positioning system (APS, part of the GMPE, Heidelberg Engineering) was used, which allows the OCT-A scans to be aligned to each individual Fovea-to-Bruch’s membrane opening center axis (Figure 2). To achieve this, the APS automatically identifies two fixed anatomical reference points: the center of the BMO (BMOC) and the Fovea. The evaluation grid is then placed based on an imaginary line between these two points. This ensures that it is aligned with the axons’ anatomical structure. Consequently, the same anatomical structures are used to evaluate each grid cell yielding more comparable results. Before each analysis, the scans are manually checked. The overall and sectorial (S1–S4) peripapillary VD were calculated, where S1 corresponds to the superior sector, S2 to the nasal sector, S3 to the inferior, and S4 to the temporal sector (Figure 3). The EA Tool computes the VD of each sector as the number of vessel pixels in the sector divided by the total number of pixels in that sector.

### 2.3. Statistical Methods

The statistical analysis was performed using SPSS version 28 (IBM Corp. Released 2021. IBM SPSS Statistics for Windows, Version 28.0. Armonk, NY, USA: IBM Corp.) and the pingouin package with Python 3.9 [42]. A *p*-value less than 0.05 was considered statistically significant. Since age and gender have been shown to have a significant correlation to ocular measurements, they were set as correction factors in all statistical analysis. Moreover, if a patient had two eligible eyes, both were included in the analysis and the patient ID was also used as a correction factor to account for patient-level clustering.

For the measurements of BMO-MRW, RNFL thickness in the inner ring, RNFL thickness in the middle ring, RNFL thickness in the outer ring, and mean VD, a covariance analysis was performed. A univariate general linear model was applied, where the diagnosis was set as predictor variable and gender and age as correction factors. Moreover, the interactions between diagnosis and age and between diagnosis and gender were modeled. If they turned out to be non-significant, these interactions were removed from the model, and the model was run again without them. Type III sum of squares was used. Moreover, the pairwise comparisons based on the estimated marginal means were computed. They were adjusted with Bonferroni to account for the multiple comparisons. For the sectorial vessel density, the procedure is basically the same as described above, except that instead of the univariate general linear model, a linear mixed model was employed. The model was modeled with a random intercept to account for the clustering in patients, and the four sectors were set as the repeated measures with a compound symmetry covariance structure. For both setups, two models were conducted: first, the diagnosis was set as a class variable with four levels (control, OHT, OAG, and pre-POAG). In the second model, the OAG was further subdivided into POAG and SOAG, leading to a total of five levels in order to investigate whether there is a difference between these two subgroups.

## 3. Results

### 3.1. BMO-MRW

BMO-MRW morphometric data of group and subgroups are presented in Table 2 with mean and standard deviation.

In the first model, age exhibited a significant effect on BMO-MRW (*p* < 0.0001), whereas gender did not (*p* > 0.05). This pattern was confirmed in the second model, where age again demonstrated a significant influence (*p* < 0.0001), while gender remained non-significant (*p* = 0.083). Considering the age effect, the Type III SS test resulted in a significant difference in BMO-MRW between diagnoses in both models (*p* < 0.0001). Table 3 displays the significant differences in BMO-MRW values for pairwise comparisons of the subgroups.

### 3.2. RNFL Thickness

Mean and standard deviation for RNFL thickness test results for all three RNFL rings (inner, middle, outer) for each subgroup are shown in Table 4.

Regarding the RNFL thickness, the first model revealed a significant gender effect for the inner (*p* = 0.11), middle (*p* = 0.010), and outer ring (*p* = 0.046). The second model confirmed this finding, showing a significant gender dependence across all three rings (*p* < 0.05). An age effect of RNFL thickness was also observed in the first model for the inner (*p* = 0.002), middle (*p* = 0.007), and outer ring (*p* = 0.012); similarly, the second model demonstrated a significant age effect for the inner (*p* = 0.002), middle (*p* = 0.008), and outer ring(*p* = 0.013). Considering the age and gender effects, Type III SS test yielded a significant effect of diagnosis on RNFL of the inner (*p* < 0.0001), middle (*p* < 0.0001), and outer ring (*p* < 0.0001) for the first model. Similarly, the Type III SS test revealed a significant diagnostic effect across all three rings in the second model (*p* < 0.0001). Table 5 shows the significance values in both models of the respective RNFL thickness of the inner, middle, and outer layers when comparing the subgroups pairwise.

### 3.3. Visual Field Index Mean Defect

Mean and standard deviations of the mean defect (MD) of OHT, pre-perimetric OAG, POAG, and SOAG are shown in Table 6.

A significant correlation was found between APSified-BMO-based-peripapillary VD and MD in OHT (*p* = 0.005), POAG (*p* = 0.035) and SOAG (*p* = 0.004), but not in pre-perimetric-OAG (*p* = 0.052), respectively.

### 3.4. Spherical Equivalent

Mean and standard deviations of the spherical equivalent of OHT, pre-perimetric OAG, POAG, and SOAG are shown in Table 7.

No significant correlation was found between APSified-BMO-based peripapillary VD and spherical equivalent in any cohort group (*p* > 0.05).

### 3.5. Vessel Density

Mean and standard deviations of overall and sectorial APSified-BMO-based peripapillary VD of OHT, pre-perimetric OAG, POAG, SOAG, and controls are shown in Table 8.

Mean APSified-BMO-based peripapillary VD demonstrated a significant correlation with age in both models (*p* < 0.0001). No gender effect was observed in either the first or the second model (*p* > 0.05). After accounting for the age effect in subsequent analyses, mean APSified-BMO-based peripapillary VD was significantly associated with diagnosis in both models (*p* < 0.0001). Table 9 shows the significant differences in APSified-BMO-based peripapillary VD for pairwise comparisons of each subgroup for the first analysis and for the OAG subanalysis (model 2).

In view of a possible variability of the APSified-BMO-based peripapillary VD due to its localization, Type III fixed-effects tests revealed a significance between sector and APSified-BMO-based peripapillary VD (*p* < 0.0001). A statistical significance (*p* < 0.0001) was also shown by the interaction between diagnosis and sector. Gender had no effect on sectorial APSified-BMO-based peripapillary VD (*p* > 0.05), whereas age had a significant effect on sectorial APSified-BMO-based peripapillary VD (*p* < 0.0001), a finding that was consistent across both models.

In the pairwise comparisons between the sectors shown in Table 10, the different subtypes made no difference, so they have been combined into one group in the table below.

Considering the effect of age, the first model showed significant differences between OAG and controls (*p* < 0.0001) and between OAG and OHT (*p* = 0.016) in each of the four sectors. In the second model, a significant difference was found between POAG and controls (*p* = 0.001), between SOAG and controls (*p* = 0.018), and between OHT and POAG (*p* = 0.036) in each of the four sectors, respectively.

### 3.6. Receiver Operating Characteristic (ROC) for Mean VD, BMO-MRW, and RNFL

**Figure 4:**
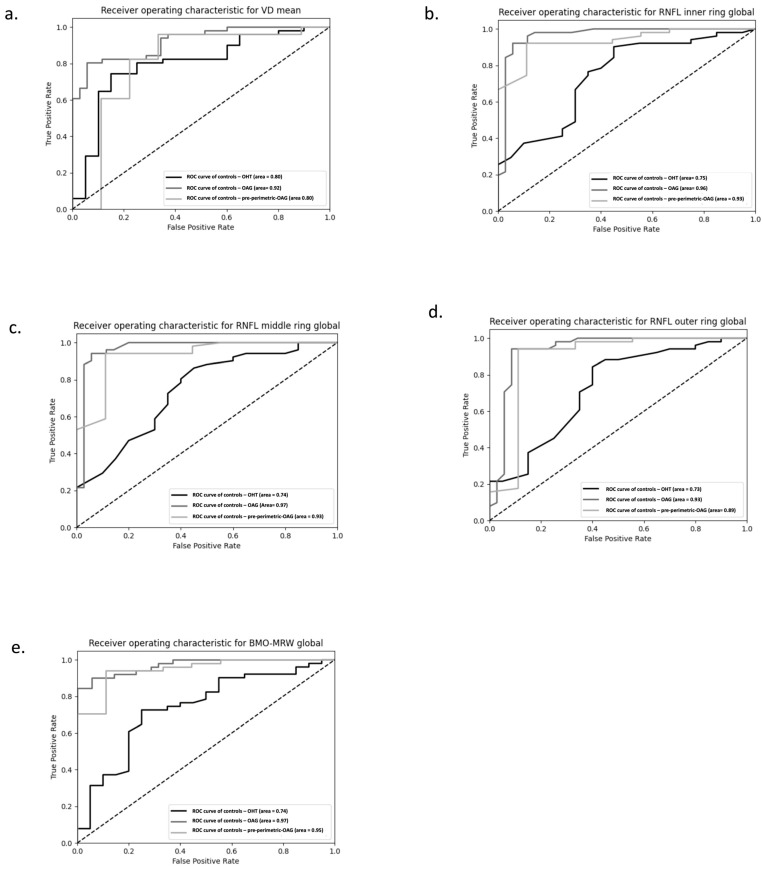
Receiver operating curves (ROC) of (**a**) mean vessel density (VD), (**b**) inner ring of retinal nerve fiber layer (RNFL) thickness, (**c**) middle ring of RNFL thickness, (**d**) outer ring of RNFL thickness, and (**e**) the Bruch’s Membrane Opening–Minimum Rim Width (BMO-MRW) are used to differentiate between the individual patient groups (ocular hypertension (OHT), pre-perimetric open-angle glaucoma (pre-perimetric OAG), primary OAG (POAG), and secondary OAG (SOAG)) and the controls. The dashed line shows the random classifier (AUC = 0.5).

ROC analyses were performed for the mean VD, the RNFL (inner, middle and outer ring), and the BMO-MRW (Figure 4). The ROCs for VD, RNFL, and BMO values demonstrate that all three groups (OHT, OAG, and pre-perimetric OAG) perform better than a random classifier in this model.

## 4. Discussion

Glaucoma is a progressive optic neuropathy with a pathological impact on microcirculation. It is evident that reduced blood flow and capillary dropout precede the onset of visual field loss in glaucoma [43,44,45]. A recent study showed that an impaired macular microcirculation was observed even in eyes of glaucoma suspects, suggesting that restricted microcirculation is an early factor in glaucoma pathology. Previous data showed that peripapillary microcirculation parameters exceed those of the macula, and therefore, the peripapillary region is suggested to show a high discriminatory power for the classification of glaucoma [46,47]. As peripapillary VD is usually quantified in ring-shaped or elliptical-shaped regions of interest, it was the aim of the present study to investigate BMO-based VD quantifications in glaucoma suspects and patients with glaucoma compared to controls. The data of the present study showed that APSified-BMO-based peripapillary VD also worsened with age and showed no gender effect. RNFL thickness and BMO-MRW decreased progressively with age and showed a high correlation with diagnosis. A significant reduction in APSified BMO-based peripapillary VD was observed in eyes with OAG compared to controls, yet not in glaucoma suspects. The present study shows an age difference between the control group and the groups with glaucoma and suspected glaucoma. As age affects peripapillary VD and decreases with increasing age, which could influence the results, we included age as a covariate in our statistical analysis to minimize its impact [48].

OCT-A is an imaging technology, enabling a non-invasive visualization of high-resolution 3D images of the retinochoroidal microcirculation in the macular and peripapillary regions. A motion contrast algorithm is used to detect moving blood cells, while the temporal diffuse reflection (due to the movement of blood cells) in the same region allows a quantification of the capillary microcirculation. Due to the differing scan sizes and lack of standardized analysis software across different OCT-A devices, different vessel density data were and will be generated. In addition, inter-individual anatomical variations must be considered, as these can have an impact on regional analysis. Implementing the APS and BMO tools might allow an enhanced inter- and intra-individual analysis. The implementation of BMO-based analysis allows an individual ONH-adapted peripapillary analysis of the VD. Recent studies defined margins of the ONH manually or automatically with rings of different sizes [33,34,49].

The quality of the scan has also been shown to influence VD values, with an improvement in scan quality correlating with an increase in peripapillary VD values [50]. Therefore, scans that were marked by an expert ophthalmologist as not good enough to use in clinical routine were removed from the study. In our study, all scans were performed with the Heidelberg Spectralis II OCT, which provides an in-depth visualization of the capillary vasculature. It features a probabilistic, full-spectrum amplitude decorrelation algorithm. This reduces the signal-to-noise ratio and improves axial resolution [24]. The use of a projection artifact removal (PAR) algorithm results in the elimination of projections of higher-lying vessels in deeper layers. The Erlangen Angio Tool was used for the calculation of the vessel density. This tool has been proven to be reliable and reproducible in a clinical study setting [41].

Previous studies have demonstrated a reduction in peripapillary VD in glaucomatous eyes when compared to age-matched normal eyes [51,52,53,54]. These findings are consistent with those observed in our own study. Furthermore, the pairwise comparison of the sectorial APSified-BMO-based peripapillary VD of all subgroups revealed significant differences, except for pairwise comparison of Sector 1 against Sector 3 and Sector 3 against Sector 4, arguing for a distinct role of regional microcirculation. The extent of VD reduction and RNFL thinning varies in different sectors, which makes a sectoral investigation a valuable approach. The study by Pradhan et al. indicates that the temporal sector exhibits reduced vessel density with normal RNFL thickness, arguing that OCT-A changes may precede RNFL changes in some sectors [55]. Moreover, several studies have shown that the diagnostic accuracy for detecting glaucoma outperformed the peripapillary region in comparison to the macular region [46,47,56]. In a longitudinal study examining the progression of glaucoma risk factors, lower baseline macular and peripapillary VD were associated with faster retinal nerve fiber layer (RNFL) progression in mild forms of glaucoma. This suggests that OCT-A may provide valuable information for the assessment of glaucoma risk factor progression and the prediction of disease worsening [57].

The VD also varies significantly depending on the OCTA device used [58,59]. These differences may be due to variations in hardware characteristics, scanning speed, segmentation strategy, and the various algorithms different manufacturers use to quantify the data [60,61,62,63]. Consequently, VD data from different devices is often not directly comparable [64]. In addition to the device itself, imaging parameters such as scan size can influence the measured vascular data [65]. Larger scan areas usually result in lower VD values than smaller, higher-resolution scans due to lower spatial sampling [66]. In this study, all measurements were performed using the same OCTA device and scan size to ensure methodological consistency; consequently, the results may not be generalizable. Therefore, the data may not be transferable to other devices and setups

Contrary to the data of the present study of peripapillary VD, a significant difference in macular VD was identified between the OHT group and controls [21]. This phenomenon may be explained by the blood supply to the ONH: The outer part of the retina is supplied by the choroid, which is regulated by the autonomic nervous system; the inner part of the retina is nourished by different plexus of the central retinal artery, showing an autoregulation the superficial plexus, supplying the nerve fiber layer and ganglion cell layer; the intermediate plexus supplyies the inner plexiform layer and the deep capillary plexus with nutrition of the outer plexiform layer [67,68,69,70,71]. The ONH, in particular, is supplied by a fourth capillary plexus. There is evidence that the autoregulation of the ONH is still intact in eyes with OHT yet impaired in OAG [72,73].

The present study is not without limitations. First, we have a European study cohort, and since ethnicity plays a role in retinochoroidal microcirculation, the present data should not be considered as overall data. Furthermore, an age trend was observed, being integrated as a covariable in the statistical analysis. Further research is necessary, including age-matched controls and a longitudinal follow-up. Additionally, despite the significant differences between the groups, there is high variability in the measured APSified-BMO-based peripapillary VD between subjects. This reduces the significance of analyzing individual subjects, particularly those from the pre-perimetric OAG group.

## 5. Conclusions

APSified-BMO based VD analysis enables a quantification of retinochoroidal microcirculation based on fixed anatomical borders. As APSified-BMO based peripapillary VD was significantly decreased in glaucoma patients compared to controls, yet not in glaucoma suspects, the data of this cross-sectional study might indicate a potential follow-up marker. Nevertheless, as this is a cross-sectional study, longitudinal studies are required in order to accurately evaluate its potential use in glaucoma progression monitoring.

## Figures and Tables

**Table 1:** Demographic data of the study cohort: number of eyes, age and gender; ocular hypertension (OHT); pre-perimetric open-angle glaucoma (pre-perimetric OAG); primary OAG (POAG); secondary OAG (SOAG).

		Number of Eyes	Age (Years)	Gender (Female/Male)
OHT		35	61 ± 15	17/18
Pre-perimetric OAG		16	66 ± 13	7/9
OAG	POAG	37	69 ± 8	23/14
	SOAG	27	68 ± 13	12/15
Controls		65	41 ± 20	42/23

**Table 2:** BMO-MRW morphometric data: Mean and standard deviation for controls, ocular hypertension (OHT), pre-perimetric open-angle glaucoma (pre-perimetric OAG), primary open-angle glaucoma (POAG) and secondary open-angle glaucoma (SOAG).

		Controls	OHT	Pre-Perimetric OAG	POAG	SOAG
BMO-MRW	Average ± SD in µm	343.44 ± 78.57	278.16 ± 70.42	207.13 ± 47.65	173.43 ± 52.89	191. 13 ± 72.56

**Table 3:** Significance of differences between BMO-MRW values in pairwise comparisons between subgroups: the first model compared controls with ocular hypertension (OHT), open-angle glaucoma (OAG), and pre-perimetric open-angle glaucoma (pre-perimetric-OAG); the second model examined pairwise comparisons for controls, OHT, pre-perimetric OAG, as well as a subanalysis of OAG, including primary open-angle glaucoma (POAG) and secondary open-angle glaucoma (SOAG).

	Model 1	Model 2
Control vs. OHT	*p* > 0.05	*p* = 1.000
Control vs. OAG	*p* < 0.0001	N/A
Control vs. Pre-perimetric OAG	*p* = 0.005	*p* = 0.008
Control vs. POAG	N/A	*p* < 0.0001
Control vs. SOAG	N/A	*p* < 0.0001
OHT vs. OAG	*p* = 0.000	N/A
OHT vs. Pre-perimetric OAG	*p* = 0.049	*p* = 0.085
OHT vs. POAG	N/A	*p* < 0.0001
OHT vs. SOAG	N/A	*p* = 0.001

(N/A = not applicable).

**Table 4:** Retinal nerve fiber layer thickness (RNFL): Mean and standard deviation for controls, ocular hypertension (OHT), pre-perimetric open-angle glaucoma (pre-perimetric OAG), primary open-angle glaucoma (POAG), and secondary open-angle glaucoma (SOAG).

			Controls	OHT	Pre-Perimetric OAG	POAG	SOAG
RNFL thickness	Inner Ring	Average ± SD in µm	99.21 ± 9.74	87.78 ± 13.66	87.73 ± 13.73	65.73 ± 10.35	69.29 ± 16.13
	Middle Ring		85.33 ± 7.99	75.14 ± 11.76	68.07 ± 11.64	58.84 ± 9.94	60.42 ± 13.08
	Outer Ring		74.53 ± 6.67	66.47 ± 9.74	60.08 ± 10.28	58.29 ± 13.77	55.39 ± 11.59

**Table 5:** Significance of RNFL thickness values for inner, middle, and outer rings in pairwise comparisons of subgroups. Model 1 compared controls, ocular hypertension (OHT), open-angle glaucoma (OAG), and pre-perimetric open-angle glaucoma (pre-perimetric OAG) pairwise. Model 2 additionally examined the subanalysis of OAG, which is divided into primary open-angle glaucoma (POAG) and secondary open-angle glaucoma (SOAG).

	Model 1			Model 2		
	Inner	Middle	Outer	Inner	Middle	Outer
Control vs. OHT	*p* = 1.000	*p* = 0.705	*p* = 1.000	*p* = 1.000	*p* = 1.000	*p* = 1.000
Control vs. OAG	*p* < 0.0001	*p* < 0.0001	*p* < 0.0001	N/A		
Control vs. Pre-perimetric OAG	*p* < 0.0001	*p* < 0.0001	*p* = 0.001	*p* = 0.001	*p* < 0.0001	*p* = 0.002
Control vs. POAG	N/A			*p* < 0.0001	*p* < 0.0001	*p* < 0.0001
Control vs. SOAG	N/A			*p* < 0.0001	*p* < 0.0001	*p* < 0.0001
OHT vs. OAG	*p* < 0.0001	*p* < 0.0001	*p* < 0.0001	N/A		
OHT vs. Pre-perimetric OAG	*p* = 0.013	*p* = 0.007	*p* = 0.027	*p* = 0.021	*p* = 0.011	*p* = 0.046
OHT vs. POAG	N/A			*p* < 0.0001	*p* < 0.0001	*p* < 0.0001
OHT vs. SOAG	N/A			*p* < 0.0001	*p* < 0.0001	*p* < 0.0001

(N/A = not applicable).

**Table 6:** Mean defect: Mean and standard deviation for ocular hypertension (OHT), pre-perimetric open-angle glaucoma (pre-perimetric OAG), primary open-angle glaucoma (POAG), and secondary open-angle glaucoma (SOAG).

		OHT	Pre-Perimetric-OAG	POAG	SOAG
Visual field mean deviation	Average ± SD in dB	2.83 ± 3.34	2.95 ± 2.26	8.43 ± 5.47	6.45 ± 4.55

**Table 7:** Spherical equivalent: Mean and standard deviation for controls, ocular hypertension (OHT), pre-perimetric open-angle glaucoma (pre-perimetric OAG), primary open-angle glaucoma (POAG), and secondary open-angle glaucoma (SOAG).

		Controls	OHT	Pre-Perimetric OAG	POAG	SOAG
Spherical equivalent	Average ± SD	−0.5 ± 2	−0.7 ± 3	−1.1 ± 3	−0.8 ± 2	−1.1 ± 3

**Table 8:** APSified-BMO-based peripapillary vessel density (VD): Mean and standard deviation for controls, ocular hypertension (OHT), pre-perimetric open-angle glaucoma (pre-perimetric OAG), primary open-angle glaucoma (POAG), and secondary open-angle glaucoma (SOAG).

			Controls	OHT	Pre-Perimetric OAG	POAG	SOAG
APSified-BMO-based peripapillary VD	Sector 1	Average ± SD	37.58 ± 7.67	33.23 ± 6.94	34.61 ± 14.96	26.06 ± 7.76	25.34 ± 9.71
	Sector 2		42.32 ± 9.77	37.11 ± 7.35	38.51 ± 12.11	32.26 ± 9.13	31.21 ± 10.99
	Sector 3		39.99 ± 5.59	36.78 ± 7.01	36.91 ± 12.55	27.27 ± 7.29	27.09 ± 8.21
	Sector 4		42.19 ± 8.64	36.42 ± 10.57	38.31 ± 13.29	30.70 ± 10.68	28.49 ± 10.52
	Mean		40.27 ± 6.76	35.89 ± 6.73	37.08 ± 12.47	29.07 ± 7.37	28.03 ± 8.66

**Table 9:** Significance of APSified-BMO-based peripapillary vessel density (VD) values in pairwise comparisons of subgroups: model 1 compared controls, ocular hypertension (OHT), open-angle glaucoma (OAG), and pre-perimetric open-angle glaucoma (pre-perimetric OAG) pairwise; model 2 additionally examined the subanalysis of OAG, which is divided into primary open-angle glaucoma (POAG) and secondary open-angle glaucoma (SOAG).

	Model 1	Model 2
Control vs. OHT	*p* = 1.000	*p* = 1.000
Control vs. OAG	*p* < 0.0001	N/A
Control vs. Pre-perimetric-OAG	*p* = 1.000	*p* = 1.000
Control vs. POAG	N/A	*p* = 0.001
Control vs. SOAG	N/A	*p* = 0.018
OHT vs. OAG	*p* = 0.016	N/A
OHT vs. Pre-perimetric-OAG	*p* = 1.000	*p* = 1.000
OHT vs. POAG	N/A	*p* = 0.036
OHT vs. SOAG	N/A	*p* = 0.324

(N/A = not applicable).

**Table 10:** Results of the pairwise comparison of all individual sectoral APSified-BMO-based peripapillary vessel density significance values among all subgroups (controls, ocular hypertension (OHT), pre-perimetric open-angle glaucoma (pre-perimetric OAG), primary OAG (POAG), and secondary OAG (SOAG)) were combined, as they did not differ from each other.

	Control, OHT, Pre-Perimetric OAG, POAG, SOAG
Sector 1 vs. 2	*p* < 0.0001
Sector 1 vs. 3	*p* = 0.098
Sector 1 vs. 4	*p* < 0.0001
Sector 2 vs. 3	*p* < 0.0001
Sector 2 vs. 4	*p* = 0.017
Sector 3 vs. 4	*p* = 0.241

## Data Availability

The datasets generated and/or analyzed during the current study are available from the corresponding author, B.H., on reasonable request.
